# Skeletal muscle insulin signaling and whole‐body glucose metabolism following acute sleep restriction in healthy males

**DOI:** 10.14814/phy2.13498

**Published:** 2017-12-12

**Authors:** Emma L. Sweeney, Stewart Jeromson, D. Lee Hamilton, Naomi E. Brooks, Ian H. Walshe

**Affiliations:** ^1^ Physiology, Exercise and Nutrition Research Group University of Stirling Stirling Scotland United Kingdom; ^2^ Department of Sport, Exercise and Rehabilitation Faculty of Health and Life Sciences Northumbria University Newcastle upon Tyne United Kingdom

**Keywords:** Glucose tolerance, metabolism, peripheral insulin signaling, PKB activity

## Abstract

Sleep restriction is associated with impaired glucose metabolism and insulin resistance, however, the underlying mechanisms leading to this impairment are unknown. This study aimed to assess whether the decrease in insulin sensitivity observed after sleep restriction is accompanied by changes in skeletal muscle PKB activity. Ten healthy young males participated in this randomized crossover study which included two conditions separated by a 3‐week washout period. Participants underwent two nights of habitual sleep (CON) and two nights of sleep which was restricted to 50% of habitual sleep duration (SR) in the home environment. Whole‐body glucose tolerance and insulin sensitivity were assessed by an oral glucose tolerance test after the second night of each condition. Skeletal muscle tissue samples were obtained from the vastus lateralis to determine PKB activity. Findings displayed no effect of trial on plasma glucose concentrations (*P* = 0.222). Plasma insulin area under the curve was higher after sleep restriction compared to the control (*P* = 0.013). Matsuda index was 18.6% lower in the sleep restriction (*P* = 0.010). Fold change in PKB activity from baseline tended to be lower in the sleep restriction condition at 30 min (*P* = 0.098) and 120 min (*P* = 0.087). In conclusion, we demonstrated decreased whole‐body insulin sensitivity in healthy young males following two nights of sleep restriction. Skeletal muscle insulin signaling findings are inconclusive and require further study to examine any potential changes.

## Introduction

Reduced sleep is associated with a number of disorders related to health (Steptoe et al. 2006; Cappuccio et al. 2010), with the relationship between sleep restriction and impaired metabolism being well established (Morselli et al. 2010). Evidence shows that chronic short sleep duration increases the risk of developing cardiovascular diseases (Sabanayagam and Shankar 2010) as well as metabolic disorders (Gottlieb et al. 2005). Furthermore, acute sleep restriction studies have demonstrated impaired glucose clearance and decreased glucose effectiveness (Steptoe et al. 2006; Buxton et al. 2010; Donga et al. 2010; Schmid et al. 2011). Reduced glucose clearance following sleep restriction has been previously shown to be due to impaired insulin sensitivity (Buxton et al. 2010; Donga et al. 2010; Broussard et al. 2012; Rao et al. 2015; Wang et al. 2016) which can be reduced by up to 25% after only a single night of sleep restriction (Donga et al. 2010), or by restricting sleep by only 1–3 h for 3 consecutive nights in young healthy adults (Wang et al. 2016). Collectively, these data demonstrate that even acute mild sleep restriction may be detrimental to both glucose regulation and insulin sensitivity.

Many studies have shown an impairment in whole‐body glucose metabolism following sleep restriction, however, less is known about the mechanisms responsible for this impairment in glucose regulation. Recent work suggests the impairment may be the result of a defect in peripheral tissues. Rao et al. (2015) assessed insulin sensitivity following sleep restriction and demonstrated that peripheral insulin sensitivity was decreased while hepatic insulin sensitivity was unchanged, indicating that a defect in the peripheral tissues may be influential in the impairment in whole‐body glucose regulation after sleep loss. Broussard et al. (2012) have demonstrated that sleep restriction influences whole‐body insulin sensitivity which is coupled with an impairment in insulin signaling in adipocytes. These data suggest that an impairment in insulin signaling in adipose tissue may contribute to the whole body impairment in glucose control. However, while adipose tissue is an active component of glucose regulation, it is likely that other peripheral tissues, such as skeletal muscle may contribute to glucose control. Skeletal muscle accounts for up to 80% of glucose clearance under insulin‐stimulated conditions (Thiebaud et al. 1982). Therefore, it is possible that any actions of sleep restriction on insulin signaling within skeletal muscle may contribute to the impairments in whole‐body glucose homeostasis.

Protein kinase B (PKB), also known as Akt, plays an important role in the insulin signaling pathway which can influence glucose uptake in skeletal muscle and adipose tissue. PKB is activated by the insulin receptor substrate 1 (IRS1) through PtdIns 3‐kinase and phosphorylation of 3‐phosphoinositide‐dependant kinase (PDK1). Activation of PKB then leads to GLUT4 translocation and results in glucose uptake (Hancock 2010). An impairment of PKB activity can lead to disruption of the insulin signaling pathway, thus, causing a reduced response to insulin.

To our knowledge no published studies have investigated the effects of sleep restriction on PKB activity within skeletal muscle, therefore, we sought to address this gap in the literature. The aims of the present study were to (1) investigate the effects of acute sleep restriction on whole‐body glucose tolerance and insulin sensitivity and (2) investigate if any changes in whole‐body insulin sensitivity are accompanied by changes in PKB activity in skeletal muscle following acute sleep restriction. We hypothesized that whole‐body glucose tolerance and insulin sensitivity would decrease and PKB activity within skeletal muscle would be impaired following two nights of partial sleep restriction.

## Methods

### Study design

Ten participants were recruited by advertisement from the university and local area to take part in this randomized crossover trial. The study involved a prescreening visit followed by two main trials, which were separated by a 3‐week washout period. Inclusion criteria included healthy males aged 18–40 years who were nonsmokers with a normal regular sleeping pattern, defined as 7–9 h of sleep each night at approximately the same time of day. Individuals were excluded if they had travelled across time zones or carried out shift work in the past 4 weeks, reported any sleep disorders, scored >5 after completing the Pittsburgh Sleep Quality Index (Buysse et al. 1989), suffered from any health conditions such as inflammatory and metabolic disorders, or were taking medications that would interfere with metabolism. All volunteers were informed of the procedures and gave written consent prior to participating in the study which was approved by the University of Stirling, School of Sport research ethics committee.

### Prescreening visit

Participants arrived at the laboratory where height and body mass were recorded. Participants completed a standard health questionnaire, a morningness–eveningness questionnaire (Horne and Ostberg 1976) and the Pittsburgh Sleep Quality Index (PSQI) (Buysse et al. 1989) to assess overall health and sleep characteristics prior to the study. Each participant was advised on how to complete a 7‐day sleep diary and 3‐day food diary, which were to be completed before beginning the main trial. In conjunction, participants wore a wrist watch actigraphy monitor (GENEActiv, Activinsights Ltd., Cambridgeshire, UK) during the 7‐day period to objectively measure habitual sleep duration prior to the first entraining period.

### Entraining

Prior to each main trial, participants underwent 1 week of entraining in their home environment. During the entraining period participants were instructed continue with their normal routine activities and to go to bed and wake at the same time, daily, which was a similar time to their habitual sleeping patterns. Wrist watch actigraphy (GENEActiv, Activinsights Ltd., Cambridgeshire, UK) was used throughout the entraining period to ensure compliance.

### Trial conditions

Following the entraining period participants were instructed on their individual time to go to bed and wake in their home environment. Experimental trials consisted of two consecutive nights of either control sleep (CON), a time calculated from the mean habitual sleep duration which was determined from the actigraphy monitors and confirmed using the 7‐day sleep diary, or two nights of sleep restriction (SR), which was determined as 50% of mean habitual sleep duration. During the sleep restriction trial, participants slept the second half of the night to ensure laboratory measurements were obtained at the same time from wakening in both trials. To confirm compliance during the sleep restriction trial, participants wore wrist actigraphy monitors and sent time‐stamped SMS messages to the researcher every hour from their habitual bed time until their sleep restricted bed time.

During the trials, participants were supplied with a 48‐h diet based on their habitual food intake from the 3‐day food diary. This diet was replicated for each trial and participants were instructed to eat only the foods provided during this 48 h period. Participants were asked to continue with their normal daily routines throughout the trials, but to avoid any vigorous physical activity or consumption of caffeine or alcohol. For safety reasons, participants were collected each morning from their home by a researcher and driven to the laboratory and returned home following each laboratory visit. Participants were instructed not to operate heavy machinery or perform tasks that may risk their safety during the trial. Participants received regular phone calls from the researchers and were instructed to have a recovery night sleep following the two nights of sleep restriction.

### Laboratory visits

Following the second night of sleep restriction or control sleep, participants arrived at the laboratory in a rested and fasted state. Height and body mass were measured. Participants then rested in the supine position for 5 min before blood pressure and heart rate were measured using a digital blood pressure monitor (Hangzhou Sejoy Electronics & Instruments Co., LTD, China). A cannula was inserted into the antecubital vein of the participant's arm to allow blood samples to be drawn at regular intervals. An oral glucose tolerance test (OGTT) was then carried out. Participants were given a drink solution consisting of 82.5 g of dextrose (Myprotein, Cheshire, UK) mixed with 300 ml of water, which they were instructed to consume all at once. Blood samples were collected before (0 min) and at 15, 30, 45, 60, 90, and 120 min following consumption of the drink. Muscle tissue samples were also obtained before (0 min) and at 30 and 120 min after the drink was consumed.

### Blood collection and processing

All blood samples during the OGTT were obtained by the cannulation technique. Ten mL of blood was collected for each sample into EDTA‐treated and serum separator vacutainer tubes. After collection, serum samples were allowed to clot at room temperature for 30 min while EDTA‐treated blood samples were placed on ice, until centrifugation. All samples were centrifuged at 1780 *g* at 4°C for 10 min. Plasma was aliquoted into microtubes and stored at −70°C until analysis.

### Muscle tissue collection and preparation

Participants laid in the supine position during the OGTT while muscle biopsies were obtained from the vastus lateralis using the Bergstrom biopsy technique. Biopsies were obtained from the same limb during the OGTT with alternate limbs used in each condition. Biopsies were taken initially at the most proximal point, then approximately 3–5 cm distally for each biopsy. The site was cleaned and the local area was anesthetized by injection of 2% Lidocaine (B. Braun, Sheffield, UK). Approximately 60–100 mg of skeletal muscle tissue was obtained for each sample. Samples were immediately cleaned with a saline solution and placed into microtubes. The tissue was then snap frozen using liquid nitrogen and stored at −70°C until analysis.

### Plasma glucose and insulin assays

Plasma glucose levels were measured from EDTA‐treated blood samples collected at 0, 15, 30, 45, 60, 90, and 120 min during the OGTT. Plasma glucose was assayed in duplicate using an ILab automated analyzer (Instrumentation Laboratory, Warrington, Cheshire, UK). Serum insulin was analyzed in duplicate using ELISA techniques from a commercially available kit (Demeditec, Kiel, Germany).

### Kinase assay

Prior to conducting activity assays, skeletal muscle tissue was processed as described previously (McGlory et al. 2014). Activity assays to determine panPKB activity were then conducted as described previously (McGlory et al. 2014). Briefly, kinase assays were carried out by immunoprecipitation for 2 h at 4°C in homogenization buffer (50 mmol/L Tris·HCl pH 7.5, 0.1 mmol/L EGTA, 1 mmol/L EDTA, 1% [vol/vol] Triton X‐100, 50 mmol/L NaF, 5 mmol/L NaPPi, 0.27 M sucrose, 0.1% *β*‐mercaptoethanol, 1 mmol/L Na_3_(OV)_4_, and 1 Complete [Roche] protease inhibitor tablet per 10 mL). Activity assays for panPKB were carried out on cell lysates by IP from 300 *μ*g of cell lysate. The IP step was performed with 2 *μ*g each of PKB*α*/*β*/*γ* antibodies (DSTT, Dundee University). Antibodies were used with 2.5 *μ*L of protein G sepharose per IP to immunoprecipitate for 2 h at 4°C. The activity assay ran for 2 h.

### Statistical analysis

Data were analyzed using Minitab v.17 statistical software (Minitab Ltd., United Kingdom). Data for participant characteristics and sleep time are presented as mean ± SD. Data for plasma glucose, insulin, and muscle PKB are presented as mean ± SEM. 2 × 7 (trial × time) repeated measures ANOVA were used to analyze plasma glucose and insulin. Paired *t*‐tests were carried out post hoc to identify any differences within main effects. Data that violated the assumption of normality were transformed prior to carrying out any statistical tests. A *P* < 0.05 was used for significance. The Matsuda index (Matsuda and DeFronzo 1999) was used to calculate insulin sensitivity. Area under the curve for glucose and insulin were calculated using the trapezoidal rule. Cohen's *d* effect sizes and confidence intervals were calculated for PKB activity.

## Results

Ten males completed the study. Participant characteristics are displayed in Table 1.

**Table 1:** Participant characteristics at baseline

Age (years)	23 ± 4
Height (cm)	181.6 ± 5.8
Body mass (kg)	82.2 ± 9.5
Body mass index (kg/m^2^)	24.9 ± 2.1
Systolic blood pressure (mmHg)	125 ± 5
Diastolic blood pressure (mmHg)	70 ± 4
Habitual energy intake (kcal/day)	2369 ± 616
PSQI score	4 ± 1
Morningness–eveningness score	53 ± 8

Data presented as mean* ± *SD. *N* = 10. PSQI, Pittsburgh Sleep Quality Index; PSQI score ≥5 is indicative of poor sleep quality. Morningness–eveningness score of 16 reflective of “definite evening type” and score of 86 “definite morning type.” A score of 42–58 is indicative of “neither type.”

### Sleep characteristics

Participants’ mean habitual time to bed and wakening time were 23:44 h and 07:42 h, respectively. Mean habitual sleep duration was 484 ± 66 min. During CON and SR mean time to bed was 23:49 h and 03:19 h and mean wakening time was 07:11 h and 07:14 h, respectively. Mean sleep duration during the control and SR condition was 442 ± 78 min and 235 ± 34 min, respectively. Control sleep and habitual sleep were not significantly different (*P *=* *0.124), however, sleep duration in the SR condition was significantly shorter than in the control condition (*P *<* *0.001).

### Blood plasma variables

**Figure 1:**
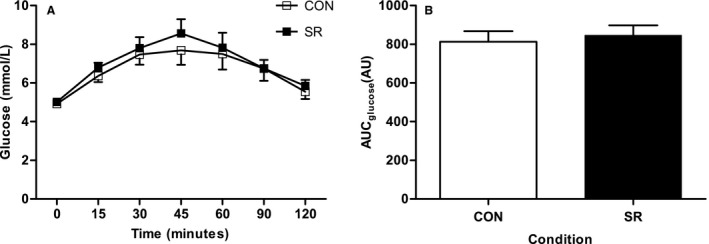
Plasma glucose concentrations. Mean plasma glucose concentrations (A) and glucose area under the curve (AUC) (B) during the OGTT following two nights of normal sleep (CON) or sleep restriction (SR) (*N* = 10). Data are expressed as mean ± SEM. Repeated measures ANOVA followed by paired *t*‐test (where appropriate) used to analyze data.

Mean plasma glucose and insulin concentrations during the OGTT are displayed in Figure 1. Repeated measures ANOVA revealed a main effect of time for plasma glucose (*P *<* *0.001). There was no effect of trial (*P *=* *0.222) or trial × time (*P *=* *0.985). Mean baseline plasma glucose concentrations during CON were similar to SR (4.92 ± 0.1 mmol/L and 5.02 ± 0.06 mmol/L, respectively) (Fig. 1A). Similarly, AUC was not different between conditions (*P *=* *0.391) (Fig. 1B).

**Figure 2:**
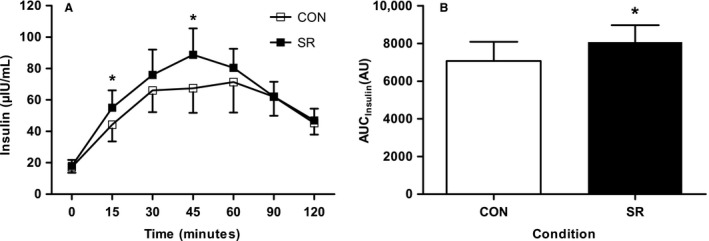
Plasma insulin concentrations. Mean plasma insulin concentrations (A) and insulin area under the curve (AUC) (B) during the OGTT following two nights of normal sleep (CON) or sleep restriction (SR) (*N* = 10). Data are expressed as mean ± SEM. Repeated measures ANOVA followed by paired *t*‐test (where appropriate) used to analyze data. * indicates significantly higher values in SR condition (*P* < 0.05).

Mean plasma insulin concentrations showed main effects of trial and time during the OGTT (*P *=* *0.006 and *P *<* *0.001, respectively). Plasma insulin values were similar at baseline in CON and SR (18.7 ± 4.9 *μ*IU/mL and 20.0 ± 5.0 *μ*IU/mL, respectively; *P *=* *0.172). SR displayed significantly higher insulin concentrations than CON at 15 min (59.1 ± 9.0 *μ*IU/mL and 48.5 ± 9.5 *μ*IU/mL for SR and CON, respectively; *P *=* *0.031) and at 45 min (83.9 ± 10.2 *μ*IU/mL and 67.3 ± 10.8 *μ*IU/mL for SR and CON, respectively; *P *=* *0.028) (Fig. 2A). AUC for insulin was 14% higher in the sleep restriction condition compared to the control (7069 ± 1018 vs. 8068 ± 901 for CON vs. SR, respectively; *P *=* *0.013) (Fig. 2B). Matsuda index was 19% lower in SR than CON (4.19 ± 0.71 vs. 3.41 ± 0.51 for CON and SR, respectively; *P *=* *0.010).

### Skeletal muscle insulin signaling

**Figure 3:**
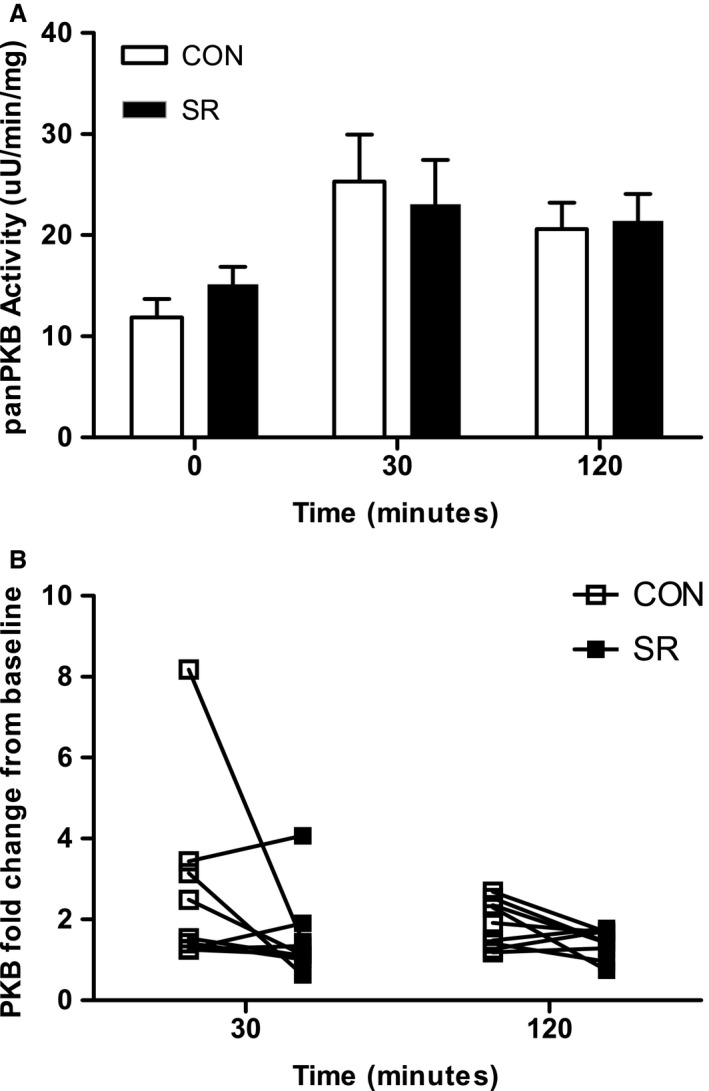
Skeletal muscle PKB activity. Skeletal muscle protein kinase B (PKB) activity during the OGTT following two nights of normal sleep (CON) or sleep restriction (SR) (*N* = 9). (A) PanPKB activity at each time point. Data are expressed as mean ± SEM. (B) Individual PKB activity fold change from baseline at 30 and 120 min.

PKB activity is shown in Figure 3. One participant was excluded from the results for PKB activity due to technical issues, therefore *N* = 9 for all PKB activity results. A main effect of time was demonstrated for panPKB activity (*P *=* *0.001) and there was a trend for trial × time (*P *=* *0.087). There was no main effect of trial (*P *=* *0.589). PanPKB activity during CON was 12.37 ± 1.93 *μ*U/(min·mg) at baseline and was significantly elevated at 30 min (26.67 ± 4.96 *μ*U/(min·mg); *P *=* *0.007) and 120 min (21.47 ± 2.75 *μ*U/(min·mg); *P *<* *0.001). PanPKB during SR was 27% higher than CON at baseline which showed a medium effect size (mean difference +3.35 *μ*U/(min·mg); 95% CI: −1.91, 8.61; d = 0.63) and showed a 16% lower response than CON at 30 min which displayed a small effect (mean difference −4.38 *μ*U/(min·mg); 95% CI: −9.60, 0.85; day = 0.32). PanPKB was similar between conditions at 120 min (21.47 ± 2.75 *μ*U/(min·mg) for CON and 21.40 ± 2.69 *μ*U/(min·mg) for SR; 95% CI: −6.38, 6.24; d = 0.01) (Fig. 3A).

PanPKB activity displayed a mean of a 2.7 fold change from baseline to 30 min in CON whereas the 1.5 fold change during SR tended to be lower (−1.13 difference in fold change; 95% CI: −2.91, 0.65; *P *=* *0.098, d = 0.69). Similarly, PKB activity showed a mean of 1.9 fold change from baseline to 120 min during CON while the 1.4 fold change during SR also tended to be lower (−0.49 difference in fold change; 95% CI: −1.03, 0.04; *P *=* *0.087, d = 1.08) (Fig. 3B).

## Discussion

The aim of the present study was to investigate if the reduction in whole‐body insulin sensitivity following acute sleep restriction was accompanied by any changes in skeletal muscle PKB activity, a protein kinase that promotes GLUT4 translocation and glucose uptake via the insulin signaling pathway. Our results demonstrate that two nights of sleep restriction impaired whole‐body insulin sensitivity; our findings regarding skeletal muscle insulin signaling following sleep restriction are inconclusive, although they do point toward potential decreases in the response of skeletal muscle PKB activity to glucose intake in healthy young men.

Plasma glucose concentrations were not altered following sleep restriction in our study. Baseline glucose values were similar between SR and CON; and furthermore, the plasma glucose response during the OGTT did not significantly differ between the conditions. This finding is in agreement with some previous studies (Zielinski et al. 2008; Schmid et al. 2009; Wang et al. 2016). However, differences have also been observed (Spiegel et al. 1999; Nedeltcheva et al. 2009; Buxton et al. 2010; Reynolds et al. 2012; Rao et al. 2015). We speculate that the differences between studies may be due to the severity of sleep loss. Studies that have shown differences in the plasma glucose response employed a more severe sleep restriction protocol than the present study. Typically, previous studies have used a set duration of sleep as a control, and/or compared sleep restriction to an extended period of sleep (e.g., 10 or 12 h). A limitation to this type of protocol is the possible individual differences in habitual sleep duration. The present study used habitual sleep duration as the control condition to overcome these limitations and employed a sleep restriction protocol which was relative to habitual sleep duration to account for possible individual differences.

We demonstrated an increased plasma insulin response during the OGTT following sleep restriction when compared to the control condition, which is consistent with previous studies (Buxton et al. 2010; Donga et al. 2010; Broussard et al. 2012; Rao et al. 2015). A higher plasma insulin response in the SR condition without any changes in plasma glucose implies that the sleep restriction protocol in our study led to reduced whole‐body insulin sensitivity. This is supported by a lower Matsuda index value following SR. Wang et al. (2016) demonstrated similar findings to the present study, showing a lower Matsuda index value after three nights of sleep restriction. The mechanisms for reduced whole‐body insulin sensitivity following sleep restriction are likely to be multifactorial. Sleep restriction has been shown to lead to insulin resistance in peripheral tissues (Rao et al. 2015), including adipose tissue (Broussard et al. 2012).

We assessed the activity of PKB in skeletal muscle at baseline, 30 min, and 120 min during the OGTT. Although we did not find a statistically significant difference for PKB activity between the conditions, our results showed a propensity for a reduced change in PKB activity in response to glucose intake in the sleep restriction condition compared to the control condition. During CON, PKB activity increased 2.7‐fold 30 min following ingestion of the drink, whereas during SR the increase was only 1.5‐fold. Similarly, at 120 min there was an increase of 1.9‐fold in CON compared to only 1.4‐fold in SR. Changes in PKB following sleep restriction have been previously shown in adipose tissue by Broussard et al. (2012). Phosphorylated PKB within adipocytes was reduced following glucose infusion in a sleep restricted condition compared to normal sleep. To our knowledge the present study is the first to investigate insulin signaling within skeletal muscle tissue following sleep restriction. The mild changes in PKB activity found in the present study may reflect the acute nature of the study, indicating that two nights may not be sufficient to elicit changes of a magnitude which can be detected at the tissue level. Furthermore, differences between studies could be due to methodologies. Broussard et al. (2012) assessed insulin sensitivity using frequently sampled intravenous glucose tolerance tests (IVGTT), where as we employed an oral glucose tolerance test (OGTT). While the OGTT demonstrates ecological validity, skeletal muscle exposure to circulating insulin can vary between participants. Therefore, it would be beneficial to explore this finding further using the gold standard euglycemic–hyperinsulinemic clamp in future studies, as well as investigating more severe sleep restriction protocols.

The effect of chronic sleep restriction on skeletal muscle insulin signaling is unknown. However, based on our results, we speculate that repeated exposure to sleep restriction, leading to disruption of whole‐body insulin sensitivity and propensity to a reduced response of PKB activity, could manifest to a possible defect in the insulin signaling pathway. Previous research suggests that a chronic increase in circulating insulin can lead to impaired insulin signaling within skeletal muscle, which is thought to be the initial defect leading to insulin resistance and the development of type 2 diabetes (DeFronzo and Tripathy 2009).

The mechanisms underlying the impairment in insulin sensitivity following sleep restriction are likely to be multifactorial, including involvement of both skeletal muscle and adipose tissue among others. Sleep restriction is often accompanied by increased inflammatory markers (Vgontzas et al. 2004; Irwin et al. 2006). Inflammation has been shown to disrupt insulin signaling in skeletal muscle (Plomgaard et al. 2005). Increased concentrations of the proinflammatory cytokine TNF‐*α* can impair glucose uptake within skeletal muscle by targeting multiple steps in the insulin signaling pathway including IRS‐1 and AS160 phosphorylation (Plomgaard et al. 2005). Therefore, the possibility of inflammation as a possible mediator between sleep restriction and impaired insulin signaling should be explored.

Insulin sensitivity can be influenced by sleep, physical activity, and food intake. With this in mind, the majority of sleep restriction studies examining insulin sensitivity have been conducted in laboratory or in‐patient settings where these factors can be controlled. We chose to perform the sleep restriction protocol in the home environment. Indeed, while we attempted to control for these factors where diet was provided, physical activity and sleep were monitored using actigraphy watches, there is always a risk of poor compliance. While our actigraphy data showed very good compliance to the sleep restriction protocol, it was not possible to assess sleep architecture. Using polysomnography (PSG), we could have gained a more detailed insight of sleep experienced in each condition and assessed the relationship between certain aspects of sleep architecture and insulin sensitivity. Future studies should take these issues into account when examining the influence of sleep restriction and insulin sensitivity.

In conclusion, two nights of 50% sleep restriction leads to impaired glucose regulation in healthy males, indicated by a reduction in whole‐body insulin sensitivity. Findings on PKB activity within skeletal muscle are unclear and therefore would benefit from additional investigation, including larger sample sizes. Future research should aim to further investigate the mechanisms underlying the impairment in glucose regulation following sleep loss.

## Conflict of Interest

None declared.
